# Massive Submacular Hemorrhage Following Intravitreal Faricimab Injection for Neovascular Age-Related Macular Degeneration

**DOI:** 10.7759/cureus.99652

**Published:** 2025-12-19

**Authors:** Adriana Kaganovski, Nairi Rostomian, Eric Shrier

**Affiliations:** 1 Ophthalmology, State University of New York Downstate Medical Center, Brooklyn, USA

**Keywords:** age-related macular degeneration (amd), faricimab, retina, submacular hemorrhage, sudden vision loss

## Abstract

We report a case of massive submacular hemorrhage (SMH) in a patient with neovascular age-related macular degeneration (AMD) occurring three days after intravitreal faricimab injection. While faricimab has demonstrated a favorable safety profile and lower hemorrhagic events compared to other anti-vascular endothelial growth factor agents, SMH, in specific, is not listed among the significant adverse events in available clinical trial safety data or in postmarketing pharmacovigilance analyses. This case highlights a new severe hemorrhagic complication that can cause permanent visual damage related to faricimab even in well-monitored patients.

## Introduction

Submacular hemorrhage (SMH) is a rare but vision-threatening complication of neovascular age-related macular degeneration (AMD) and its treatment [1]. Intravitreal anti-vascular endothelial growth factor (anti-VEGF) therapy reduces the risk of hemorrhage by stabilizing choroidal neovascular membranes; however, rupture of friable vessels may still occur [2]. Faricimab, a bispecific antibody targeting both vascular endothelial growth factor A (VEGF-A) and angiopoietin-2 (Ang2), has shown efficacy with extended dosing intervals and a favorable safety profile in clinical trials [3]. Important adverse events related to faricimab are mild intraocular inflammation, retinal pigment epithelial tears, and, rarely, occlusive retinal vasculitis. Real-world pharmacovigilance analyses report retinal and vitreous hemorrhages with faricimab, but SMH is not listed among significant adverse events. We describe a case of massive SMH occurring after faricimab injection.

## Case presentation

A 78-year-old man with neovascular AMD in the right eye (OD), dry AMD in the left eye (OS), and bilateral optic nerve head drusen presented with painless vision loss OD three days after an intravitreal faricimab injection for recurrent exudation. He had previously received aflibercept every one to two months for two years with limited improvement.

**Figure 1:**
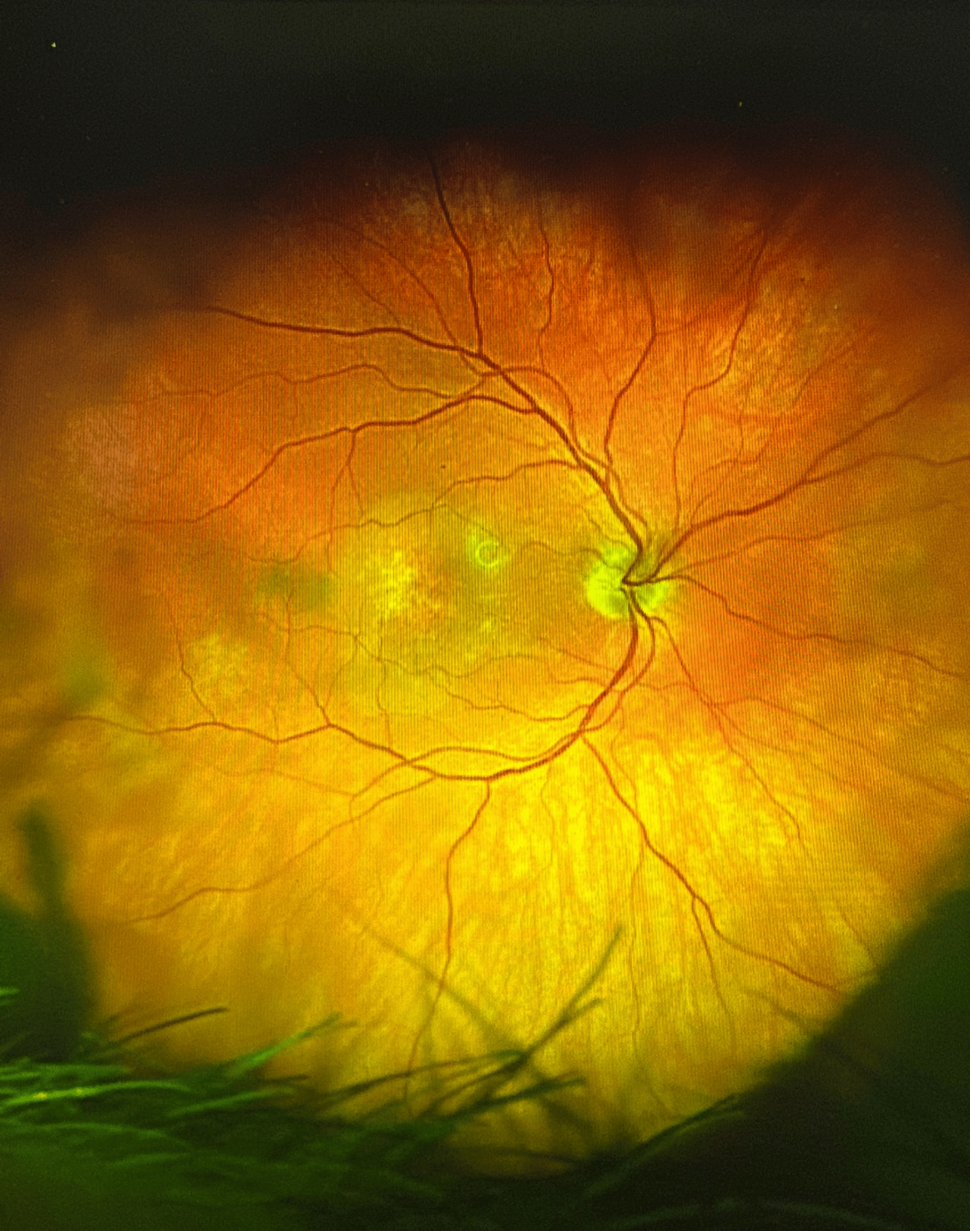
Color fundus photograph of the right eye (OD) directly prior to injection demonstrating multiple drusen and macular edema.

**Figure 2:**
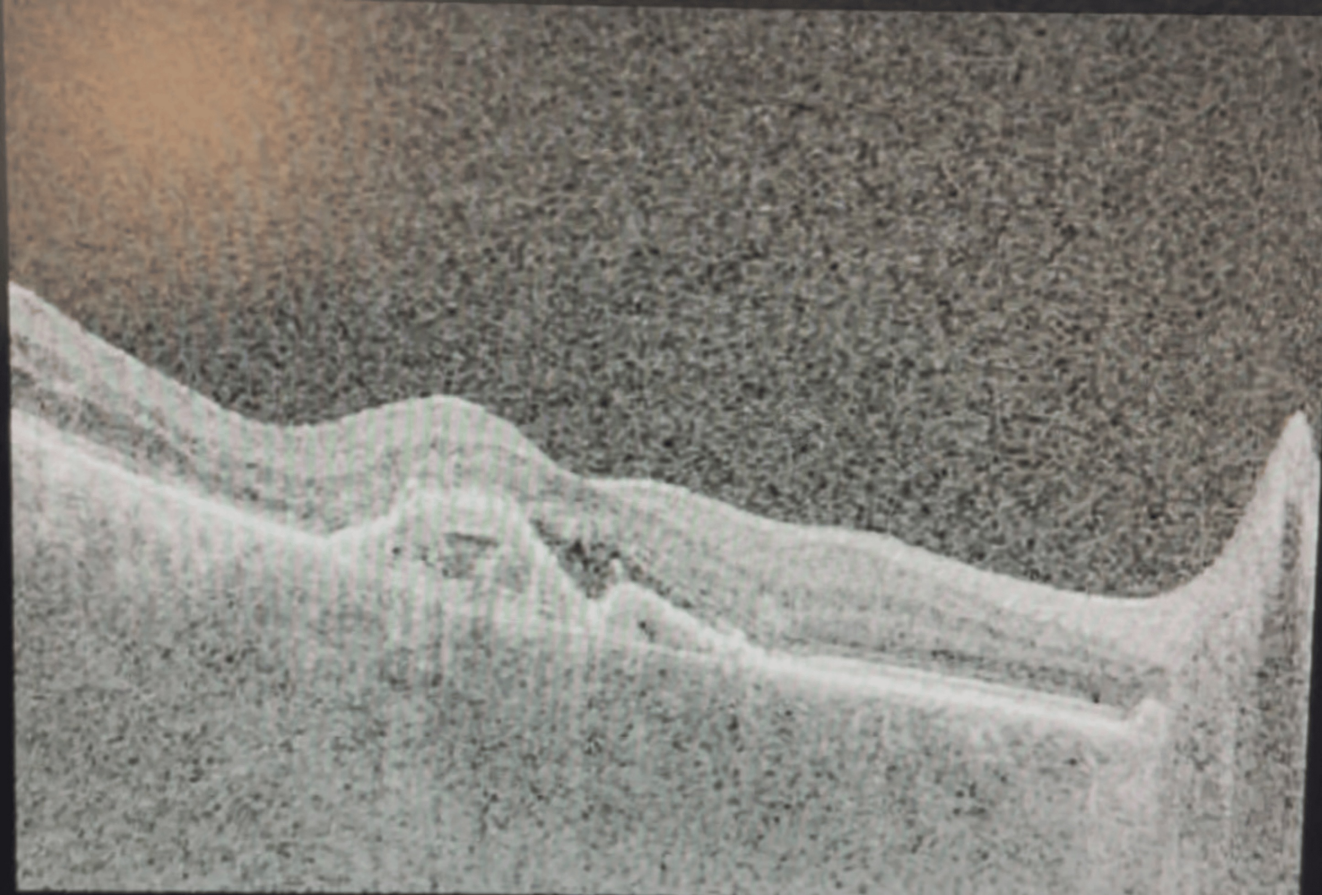
Optical coherence tomography of the right eye (OD) at baseline demonstrating one large and one medium-sized drusen with intraretinal fluid and preserved foveal contour.

Prior to faricimab, color fundus imaging showed multiple drusen and macular edema (Figure 1), and baseline optical coherence tomography (OCT) demonstrated large drusen with intraretinal fluid and preserved foveal contour (Figure 2).

**Figure 3:**
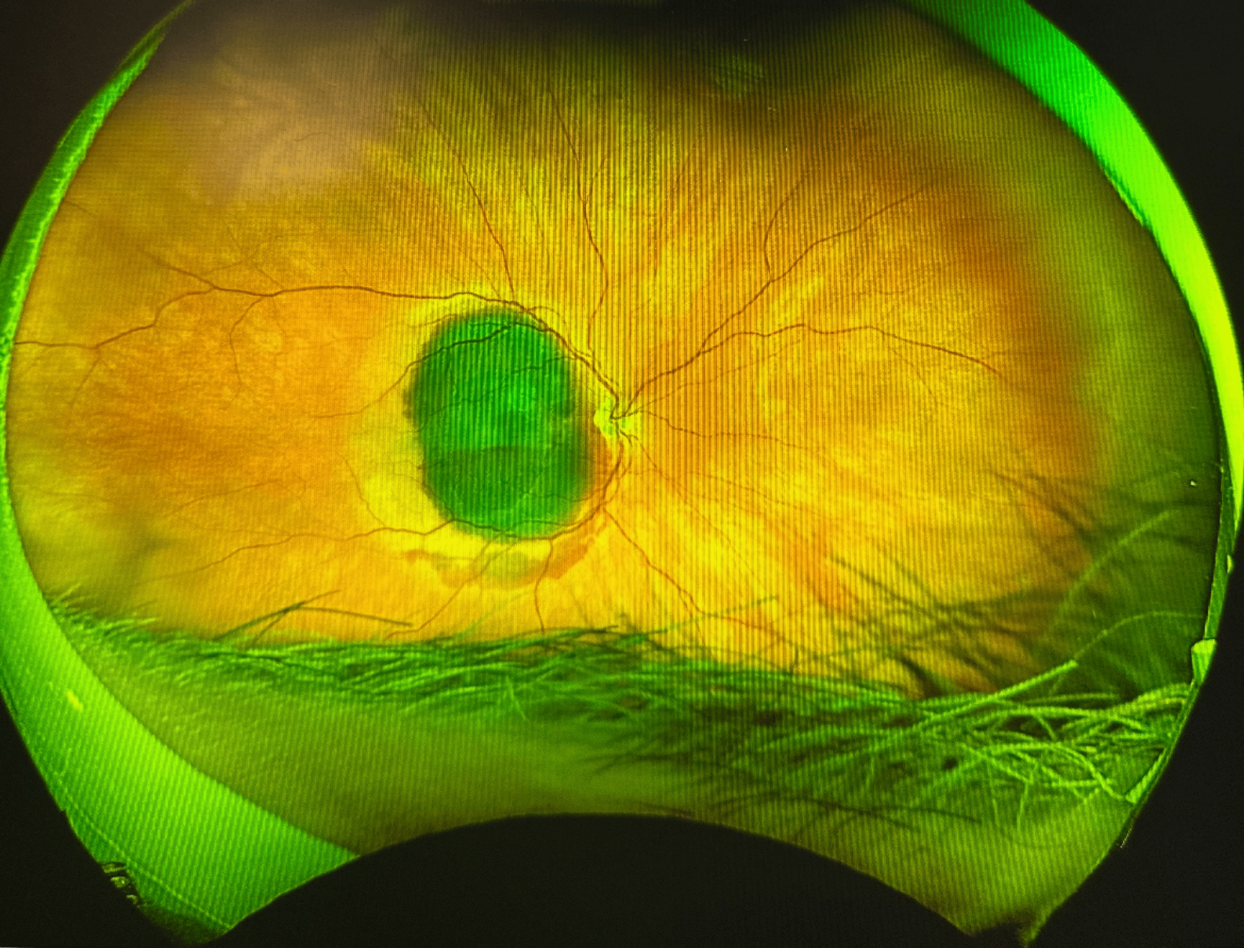
Color fundus photograph of the right eye (OD), three days after the patient presented to the ED, demonstrating a large submacular hemorrhage spanning from the superior to inferior vascular arcade.

**Figure 4:**
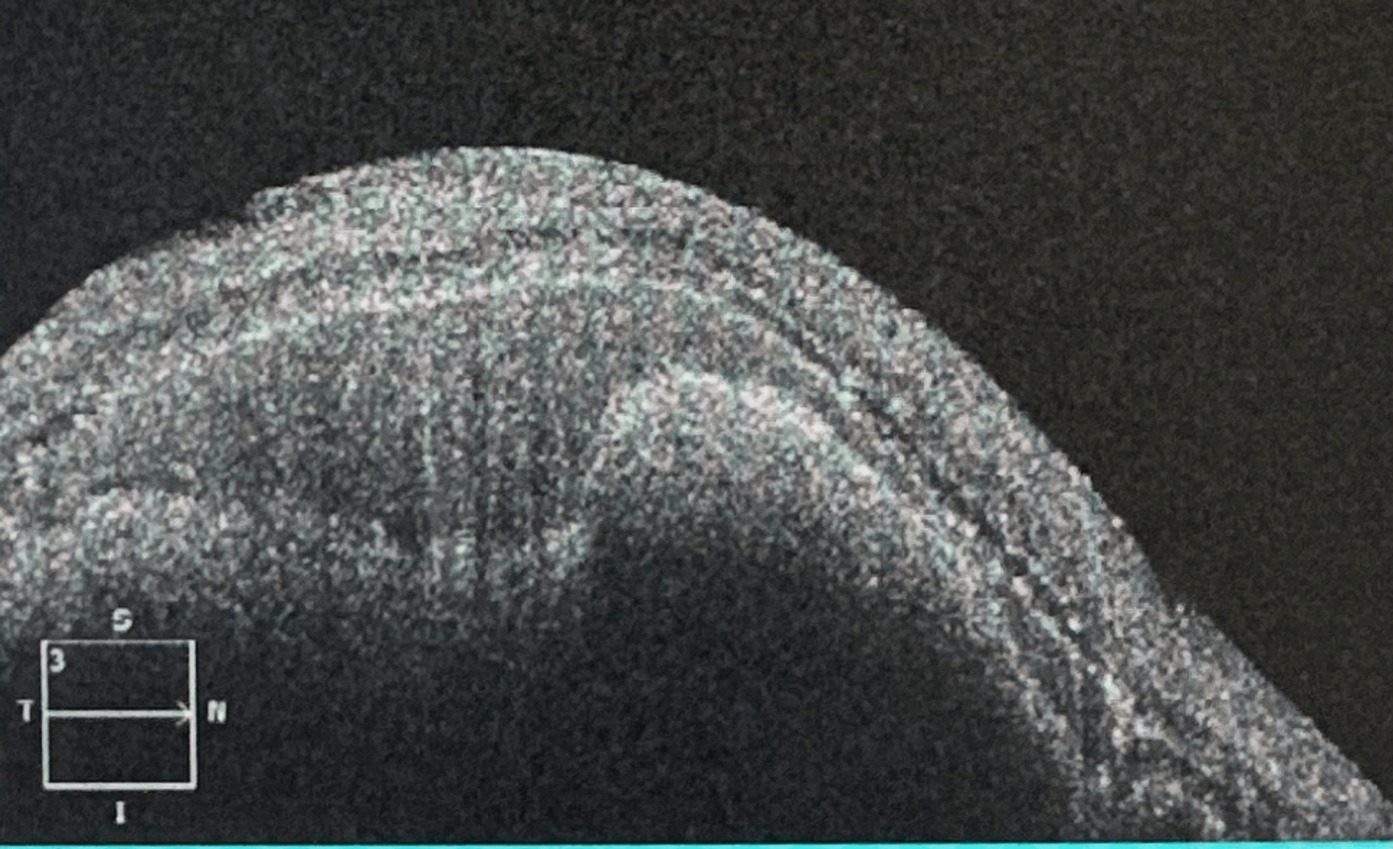
Optical coherence tomography of the right eye (OD) seven days following faricimab injection showing obliteration of the foveal contour with subretinal and developing subretinal pigment epithelium hemorrhage.

On presentation, visual acuity was counting fingers OD (previously 20/50) and 20/20 OS. Dilated fundus examination revealed a dense SMH spanning the superior to inferior arcade (Figure 3), and OCT confirmed subretinal and subretinal pigment epithelium (sub-RPE) hemorrhage with loss of foveal contour (Figure 4).

The patient was discharged with activity restrictions and returned for follow-up, where vitrectomy with tissue plasminogen activator (tPA) was deferred due to rebleeding risk. Intravitreal aflibercept was initiated.

**Figure 5:**
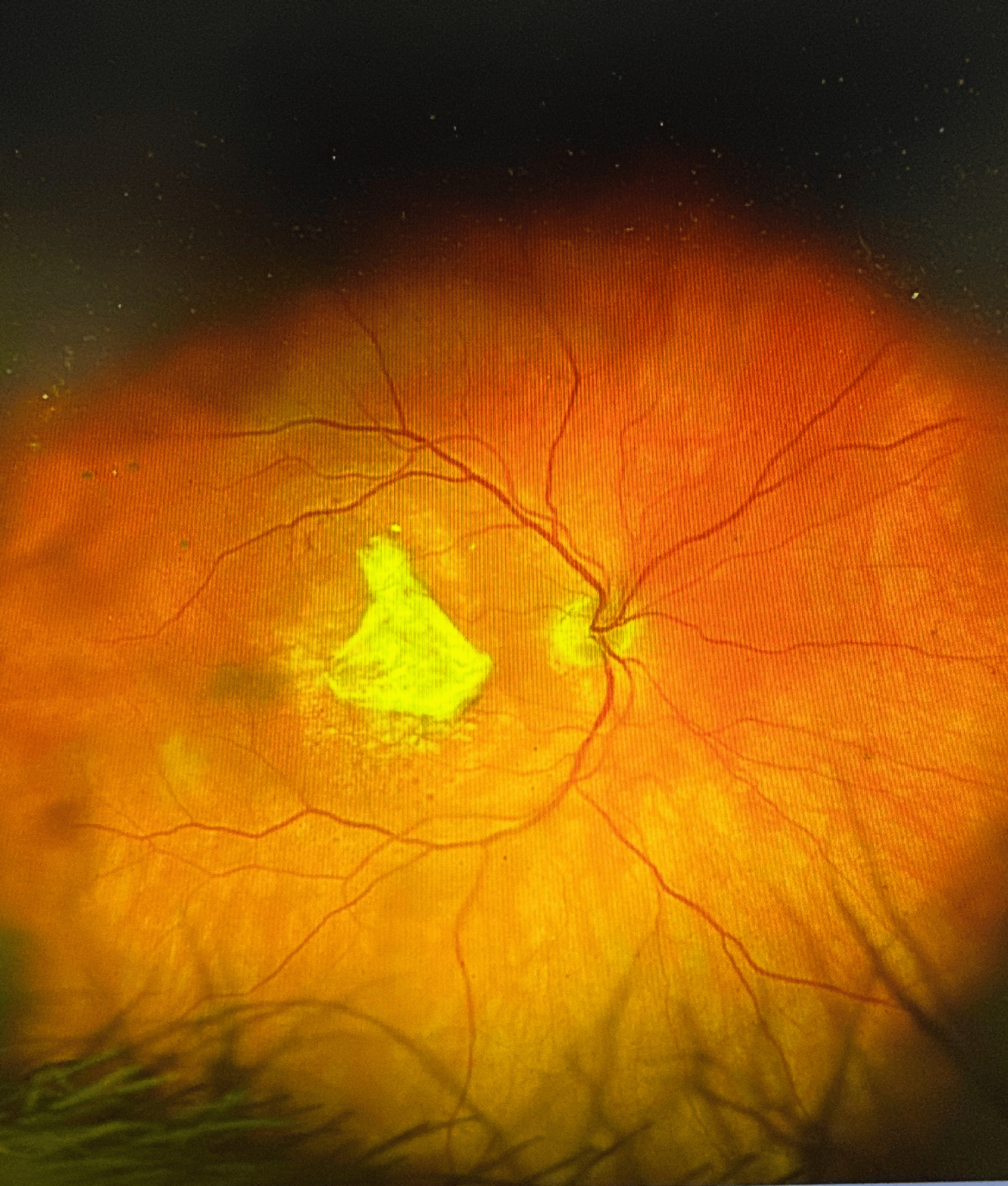
Color fundus photograph of the right eye (OD) after seven Eylea and five Eylea HD injections (27 months) demonstrates total subretinal fibrosis of the macula.

**Figure 6:**
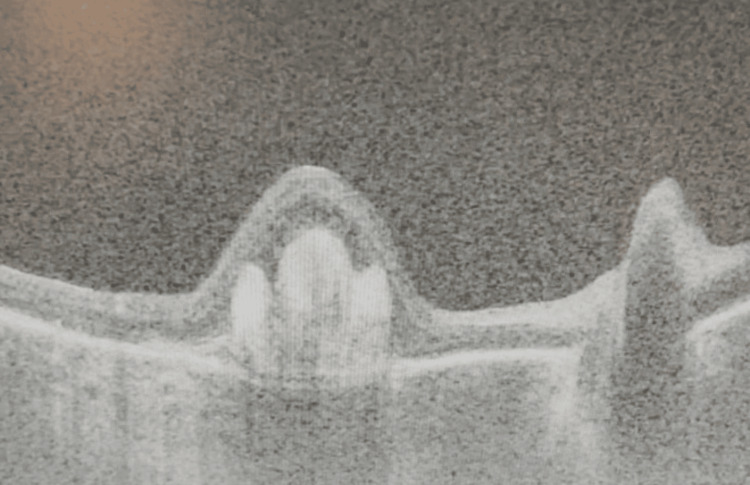
Optical coherence tomography of the right eye (OD) after seven Eylea and five Eylea HD injections (27 months) demonstrating complete resolution of hemorrhage with stable subretinal fibrosis.

At 27 months (after seven Eylea and five Eylea HD injections), vision was hand motion OD and 20/30 OS. Fundus imaging (Figure 5) and OCT (Figure 6) demonstrated complete subretinal fibrosis consistent with irreversible central vision loss.

## Discussion

Pharmacovigilance data from the U.S. Food and Drug Administration (FDA) Adverse Event Reporting System (FAERS) and the Japanese Adverse Drug Event Report (JADER) database indicate reports of retinal, vitreous, and choroidal hemorrhage with intravitreal injections of faricimab. However, SMH has not been specifically reported. This suggests that either SMH was not detected or was reported under broader hemorrhagic terms, limiting clarity on the individual incidence of such a vision-threatening complication [4,5].

In contrast, large-scale real-world evidence suggests faricimab may be protective against SMH [6]. In a cohort of more than 140,000 eyes with neovascular AMD, faricimab was associated with the lowest rate of SMH (0.21%) compared to other anti-VEGF agents, with multivariate regression confirming a protective association relative to ranibizumab even after adjusting for confounders.

Mechanistically, faricimab’s bispecific design enables simultaneous inhibition of VEGF-A and Ang2, reducing vascular leakage, reinforcing endothelial junctions through tyrosine kinase with immunoglobulin-like and epidermal growth factor-like domains 2 (Tie-2) activation, and mitigating vascular inflammation [7]. These effects promote vascular stability and may explain the researcher’s observed protective association with SMH. Taken together, these findings underscore the rarity of SMH in faricimab-treated patients, making the present case a noteworthy addition to the literature.

In this case, the patient lacked several recognized SMH risk factors, such as recent anticoagulant use or uncontrolled hypertension, but did have pre-existing friable choroidal neovascularization, a known predisposing factor for SMH [8,9]. While the temporal sequence of SMH developing just three days after intravitreal injection raises the possibility that the injection contributed to the event, it is also possible that spontaneous hemorrhage related to the natural history of neovascular AMD played a role. Therefore, a definitive causal relationship cannot be established, and this case represents a temporal association rather than proof of causality. Evidence suggests that SMHs are more likely to occur within 30 days of anti-VEGF injection [10], supporting the plausibility of an injection-related trigger, but alternative explanations remain relevant.

This case underscores the need for clinicians to remain vigilant for massive SMH following faricimab, even in patients without classic risk factors, and to counsel patients on the signs of acute hemorrhage to facilitate prompt diagnosis and intervention. The vision-threatening nature of SMH arises from its location directly beneath the macula, where blood products are toxic to the overlying photoreceptors [11]. Massive collections of blood not only cause immediate mechanical damage and separation of the photoreceptor layer but, as they resolve, they typically result in dense subretinal fibrotic scarring [12]. This process irreversibly disrupts the macular architecture and destroys the photoreceptor layer, leading to a profoundly poor visual prognosis. Consistent with this, our patient’s vision deteriorated to counting fingers, illustrating the devastating long-term impact that massive SMH can have on central vision.

## Conclusions

This case illustrates that massive SMH can occur shortly after intravitreal faricimab injection, even in patients without classic systemic risk factors for hemorrhage. Despite faricimab’s favorable safety profile and its potential protective effect against SMH reported in large-scale studies, the temporal association in this patient strongly suggests a procedure-related precipitating event. The severe and irreversible vision loss observed underscores the potentially devastating consequences of SMH and highlights the need for clinicians to recognize early signs of acute hemorrhage and promptly counsel patients regarding visual changes following injection.

Although faricimab remains an effective and generally well-tolerated therapy for neovascular AMD, this report emphasizes the importance of continued vigilance for rare but vision-threatening complications. Future pharmacovigilance efforts should aim to more specifically capture instances of SMH to better characterize its incidence and risk factors in real-world practice. Clinicians should balance the benefits of faricimab’s dual VEGF-A and Ang2 inhibition against the small but significant risk of severe hemorrhagic events and maintain individualized monitoring strategies to mitigate long-term visual morbidity.
